# Double-dose furmonertinib for patients with osimertinib-resistant advanced NSCLC: therapeutic efficacy and safety

**DOI:** 10.3389/fonc.2026.1823078

**Published:** 2026-05-08

**Authors:** Yepei Wei, Yishi Xu, Qing Ren, Yuan Zhang, Chuhan Zhang, Liping Wang

**Affiliations:** Department of Oncology, The First Affiliated Hospital of Zhengzhou University, Zhengzhou, Henan, China

**Keywords:** CNS metastasis, double-dose furmonertinib, drug resistance, lung cancer, osimertinib, patient safety

## Abstract

**Background:**

Epidermal growth factor receptor (EGFR) gene mutations play a key driving role in the development and progression of lung cancer. Currently, EGFR tyrosine kinase inhibitors (TKIs) serve as the standard first-line therapy for patients with advanced non-small cell lung cancer (NSCLC) harboring EGFR mutations. However, with the widespread use of these drugs, resistance has become increasingly prevalent, particularly with third-generation EGFR-TKIs, posing a significant bottleneck limiting survival benefits for patients. This real-world study aims to evaluate the efficacy and safety of double-dose furmonertinib in patients with advanced NSCLC who have developed resistance to prior osimertinib therapy, with the goal of offering practical insights for clinical decision-making.

**Methods:**

A retrospective analysis was conducted on 69 patients with advanced NSCLC resistant to osimertinib. The control group received platinum-based dual-agent chemotherapy, with progression leading to subsequent-line therapy. The observation group received either double-dose furmonertinib (160 mg/day, oral) or double-dose furmonertinib monotherapy in addition to the control group regimen.

**Results:**

The observation group had significantly improved disease control rate (DCR) compared with the control group (71.1% vs 41.7%, *p* < 0.05) and prolonged median progression-free survival (mPFS) (4.97 months vs 3.20 months, *p* < 0.05). Univariate Cox regression analysis indicated that treatment group (*p* = 0.021), central nervous system (CNS) metastasis (*p* = 0.012), and Eastern Cooperative Oncology Group (ECOG) performance status (*p* = 0.025) were factors influencing PFS in osimertinib-resistant advanced NSCLC patients. Multivariate analysis using the Cox proportional hazards model revealed that CNS metastasis (*p* = 0.011) and ECOG score ≥2 (*p* = 0.038) were independent risk factors affecting the prognosis of osimertinib-resistant patients. Treatment with double-dose furmonertinib (95% CI: 0.196–0.750, *p* = 0.005) was an independent protective factor for prognosis in this patient group. There were no statistically significant differences between the two groups in the overall incidence of adverse events (AEs) (66.7% vs 70.8%, *p* > 0.05) or the incidence of Grade 3–4 AEs (15.6% vs 16.7%, *p* > 0.05). No Grade 5 treatment-related AEs were monitored in this study. Neither group experienced new adverse reaction symptoms, nor did any serious fatal AEs occur.

**Conclusion:**

In patients with osimertinib-resistant advanced NSCLC, the regimen comprising double-dose furmonertinib yielded promising efficacy, accompanied by an acceptable safety profile and overall tolerable treatment-related adverse events. In patients with osimertinib-resistant advanced NSCLC and CNS metastases, double-dose furmonertinib significantly prolongs PFS and demonstrates marked local control of CNS metastases.

## 1 Introduction

Lung cancer is currently one of the most common malignant tumors worldwide and a leading cause of cancer-related deaths. Its incidence rate is extremely high and shows an upward trend year by year (1). With continuous advancements in genetic testing technology and in-depth research into tumor signaling pathways, it has been discovered that the epidermal growth factor receptor (EGFR) serves as a key oncogenic driver gene in lung cancer. Statistics indicate that approximately 50% of lung adenocarcinoma patients in China carry EGFR gene mutations (2). Therefore, in recent years, drug development targeting EGFR gene mutations has made remarkable progress in the field of cancer treatment. Currently, research in this area has yielded abundant results, with multiple drugs targeting EGFR mutations approved for market release. To date, three generations of EGFR tyrosine kinase inhibitors (TKIs) have demonstrated favorable efficacy in clinical and preclinical trials and have been approved for clinical use (3). As research has progressed, EGFR-TKIs have not only expanded treatment options for patients with advanced non-small cell lung cancer (NSCLC) but also enhanced outcomes for those with early-stage, operable NSCLC when used as neoadjuvant and adjuvant therapies (4).

Like traditional chemotherapy drugs, EGFR-TKIs also face the challenge of drug resistance. Third-generation EGFR-TKIs, including osimertinib, aumolertinib, furmonertinib, befotertinib, and lazertinib, effectively address the issue of EGFR T790M resistance mutations (5, 6). Moreover, compared to first- and second-generation EGFR-TKIs, third-generation EGFR-TKIs demonstrate more pronounced clinical efficacy, superior safety profiles, and lower rates of adverse reactions. Osimertinib, as a representative third-generation EGFR-TKI, has risen to become the recommended first-line treatment for patients with EGFR-mutated advanced NSCLC (7).

The central nervous system (CNS) is the most common site of metastasis for lung cancer. CNS metastases are more common in adenocarcinoma and large cell undifferentiated carcinoma. Studies have demonstrated that EGFR gene mutations are associated with CNS metastasis in lung cancer, with patients harboring these mutations being more susceptible to CNS spread (8). Notably, third-generation EGFR-TKIs have been shown through research to exhibit excellent blood–brain barrier permeability, demonstrating significantly greater inhibitory effects on CNS metastases compared to first- and second-generation EGFR-TKIs. Studies employing isotope tracers to label erlotinib, afatinib, and osimertinib revealed that osimertinib exhibited the highest standard uptake values in nearly all tissues following administration, particularly demonstrating the highest tracer uptake in lung, spleen, and brain tissues (9). In the FLAURA study, osimertinib demonstrated a significant progression-free survival (PFS) benefit in the CNS metastasis subgroup (10). Subsequent crossover studies demonstrated that osimertinib combined with chemotherapy exhibited superior CNS efficacy compared to monotherapy (11). The BLOOM study is a Phase I multicenter clinical trial designed to evaluate the efficacy of osimertinib in patients with advanced NSCLC who have failed prior EGFR-TKI therapy and have leptomeningeal metastasis (LM). Results demonstrated an objective response rate (ORR) of 62%, median progression-free survival (mPFS) of 8.6 months, and median overall survival (OS) of 11.0 months (12). The introduction of the trifluoroethoxy structure has demonstrated that both furmonertinib and its primary metabolite AST5902 exhibit strong blood–brain barrier penetration and tumor growth inhibitory activity. Compared to similar studies, the FURLONG trial enrolled a larger number of patients with CNS metastases. Results demonstrated that first-line use of furmonertinib in advanced NSCLC patients with classic EGFR mutations achieved an mPFS of 20.8 months. Additionally, patients with CNS metastases exhibited reduced disease progression rates and mortality risks, yielding superior outcomes compared to other drugs in the same generation (13, 14).

Despite this, resistance to third-generation EGFR-TKIs remains a significant challenge. Most advanced NSCLC patients resistant to third-generation EGFR-TKIs opt for platinum-based dual-agent chemotherapy regimens, yet the observed clinical efficacy falls far short of expectations. The safety and tolerability of platinum-based combination regimens—such as chemotherapy paired with immune checkpoint inhibitors or anti-angiogenic agents—cannot be overlooked. Consequently, exploring high-dose EGFR-TKIs emerges as a viable alternative, aiming to enhance tumor suppression by increasing the therapeutic dose of targeted agents. Thus, this study aims to retrospectively evaluate the efficacy and feasibility of double-dose furmonertinib in treating advanced NSCLC patients resistant to third-generation EGFR-TKIs (osimertinib) within a real-world clinical setting, providing evidence-based guidance for clinical decision-making.

## 2 Methods

### 2.1 Research subjects and basic information

From June 2021 to October 2024, 69 patients with EGFR-mutated advanced NSCLC who developed resistance to osimertinib treatment at the First Affiliated Hospital of Zhengzhou University were enrolled in this study. Patients were divided into two groups: the first group comprised patients who received platinum-based doublet chemotherapy administered every 21 days for four to six cycles. Patients who progressed during treatment were switched to subsequent-line therapy. The observation group comprised patients who received the control regimen plus either 160 mg of furmonertinib orally or 160 mg of furmonertinib as monotherapy. Oral medication was continued until patient intolerance or disease progression.

Clinical and pathological characteristics of patients were collected from medical records, including age, sex, Eastern Cooperative Oncology Group (ECOG) performance status, body mass index (BMI), smoking history, histological subtype, mutation sites, presence of CNS metastases, size of metastatic lesions, and TNM staging. Treatment information included other local therapies, such as surgery and radiotherapy, as well as the type of adverse reactions during treatment with 160 mg furmonertinib. The date of initiation of 160 mg furmonertinib therapy and the time to tumor progression (including time of loss to follow-up or death) were also obtained.

### 2.2 Therapeutic effect evaluation and follow-up

This retrospective, single-center, real-world study aimed to evaluate the efficacy and safety of re-administering 160 mg furmonertinib to patients with osimertinib-resistant disease following progression. The primary endpoints were PFS and ORR, with secondary endpoints including disease control rate (DCR), safety, and tolerability. Efficacy was assessed according to Response Evaluation Criteria in Solid Tumors (RECIST) 1.1. PFS was defined as the time interval from the development of osimertinib resistance to disease progression or death from any cause, whichever occurred first. ORR represents the proportion of patients achieving complete response (CR) or partial response (PR). Treatment-related adverse events (AEs) monitored during therapy were graded according to the Common Terminology Criteria for Adverse Events (CTCAE) version 5.0 and documented in detail. The last follow-up date for this study was November 30, 2024.

### 2.3 Applications of statistics

In this study, all collected data were statistically analyzed using the SPSS 26.0 software, with graphs and charts generated using the GraphPad Prism 9.5 software. For qualitative data, chi-square tests, corrected chi-square tests for continuity, or Fisher’s exact probability test were selected based on sample size. Survival analysis was performed using the Kaplan–Meier method, with corresponding survival curves plotted. The log-rank test was employed to compare survival differences between groups. Cox proportional hazards models were established for univariate and multivariate analyses to evaluate factors influencing PFS. The study significance level was set at α = 0.05.

## 3 Results

### 3.1 Baseline general clinical data

A total of 69 patients had previously received at least one cycle of osimertinib and were assessed as having PD during treatment. There were no statistically significant differences between the two groups in age, sex, ECOG performance status, BMI, smoking history, mutation site, presence of CNS metastasis, history of surgery/radiotherapy, or TNM staging (*p* > 0.05). Baseline characteristics were largely comparable. See **Table 1** for details.

**Table 1:** Baseline demographic and clinical characteristics of patients.

Variable	Observation group (n = 45)	Control group (n = 24)	*χ* ^2^	*p*-Value
Age			0.519	0.471
<65 years	26 (57.8%)	16 (66.7%)		
≥65 years	19 (42.2%)	8 (33.3%)		
Gender			0.328	0.567
Male	22 (48.9%)	10 (41.7%)		
Female	23 (51.1%)	14 (58.3%)		
BMI (kg/m^2^)			0.310	0.578
18.5–23.9	20 (44.4%)	9 (37.5%)		
<18.5 or ≥24.0	25 (55.6%)	15 (62.5%)		
Smoking history			0.513	0.474
No	30 (66.7%)	18 (75.0%)		
Yes	15 (33.3%)	6 (25.0%)		
ECOG PS			0.421	0.516
<2	28 (62.2%)	13 (54.2%)		
≥2	17 (37.8%)	11 (45.8%)		
Tumor stage			1.406	0.236
IVa	1 (2.2%)	2 (8.3%)		
IVb	44 (97.8%)	22 (91.7%)		
Pathological type			0	1.000
Adenocarcinoma	45 (100.0%)	24 (100.0%)		
EGFR status at baseline			0.821	0.365
Exon 19del	22 (48.9%)	9 (37.5%)		
Exon21 L858R	23 (51.1%)	15 (62.5%)		
Surgery/radiotherapy history			0.852	0.356
No	21 (46.7%)	14 (58.3%)		
Yes	24 (53.3%)	10 (41.7%)		
CNS metastasis			1.041	0.307
No	15 (33.3%)	11 (45.8%)		
Yes	30 (66.7%)	13 (54.2%)		

BMI, body mass index; ECOG PS, Eastern Cooperative Oncology Group performance status; EGFR, epidermal growth factor receptor; CNS, central nervous system.

### 3.2 Efficacy comparison and survival analysis

#### 3.2.1 Comparison of therapeutic efficacy between the two patient groups

**Figure 1:**
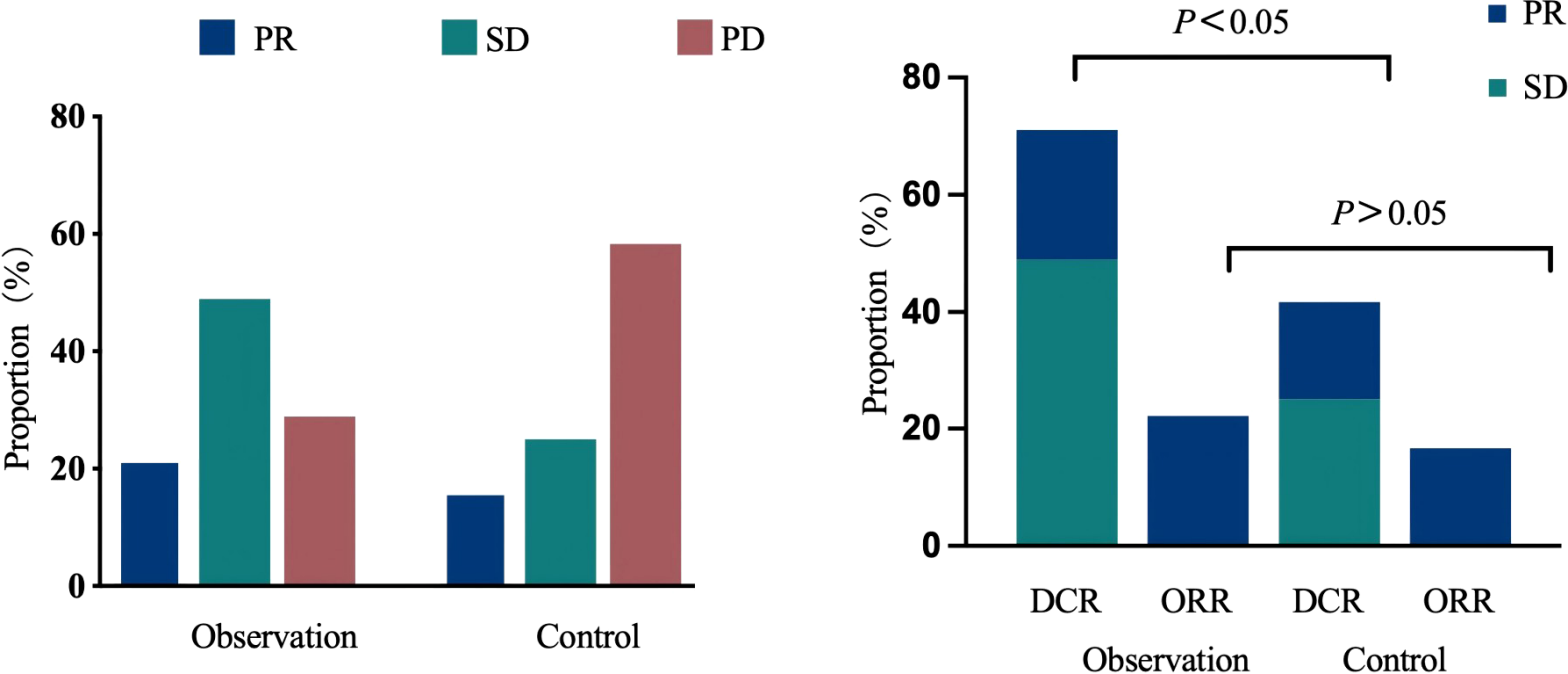
Comparison of optimal response distribution, ORR, and DCR between the two patient groups. ORR, objective response rate; DCR, disease control rate.

Efficacy was assessed in both groups according to RECIST 1.1 criteria, with no cases of CR observed in either group. In the observation group, 10 patients achieved PR, 22 patients achieved stable disease (SD), and 13 patients were assessed as progressive disease (PD), yielding an ORR of 22.2% and a DCR of 71.1%. In the control group, four patients achieved PR, six patients achieved SD, and 14 patients were assessed as PD, resulting in an ORR of 16.7% and a DCR of 41.7%. The observation group demonstrated higher ORR and DCR than the control group. The difference in DCR was statistically significant (71.1% vs 41.7%, *p* = 0.017), while the ORR difference was not statistically significant (22.2% vs 16.7%, *p* = 0.585). See **Figure 1**.

#### 3.2.2 Comparison of PFS between the two patient groups

**Figure 2:**
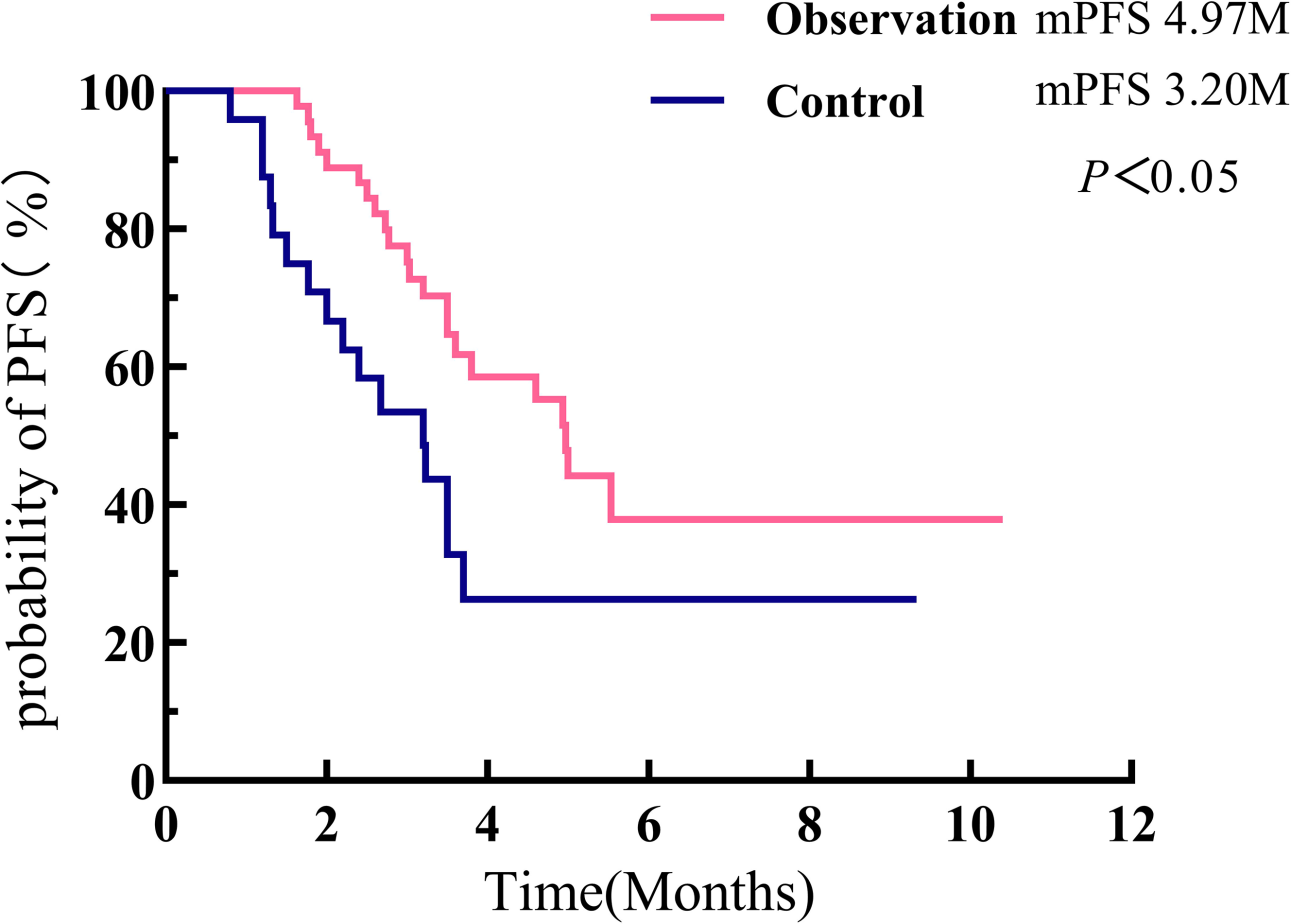
Survival curves comparing the two patient groups.

Survival analysis was performed using the Kaplan–Meier method, and the log-rank test was employed to compare whether there was a statistically significant difference in PFS between the two groups. Results showed that the mPFS in the observation group was 4.97 months (95% CI: 4.364–5.576), while that in the control group was 3.20 months (95% CI: 2.032–4.368). The mPFS in the observation group was significantly longer than that in the control group, with a statistically significant difference between the two groups (*χ*^2^ = 5.664, *p* = 0.017). See **Figure 2**.

### 3.3 Factors influencing PFS analysis

**Figure 3:**
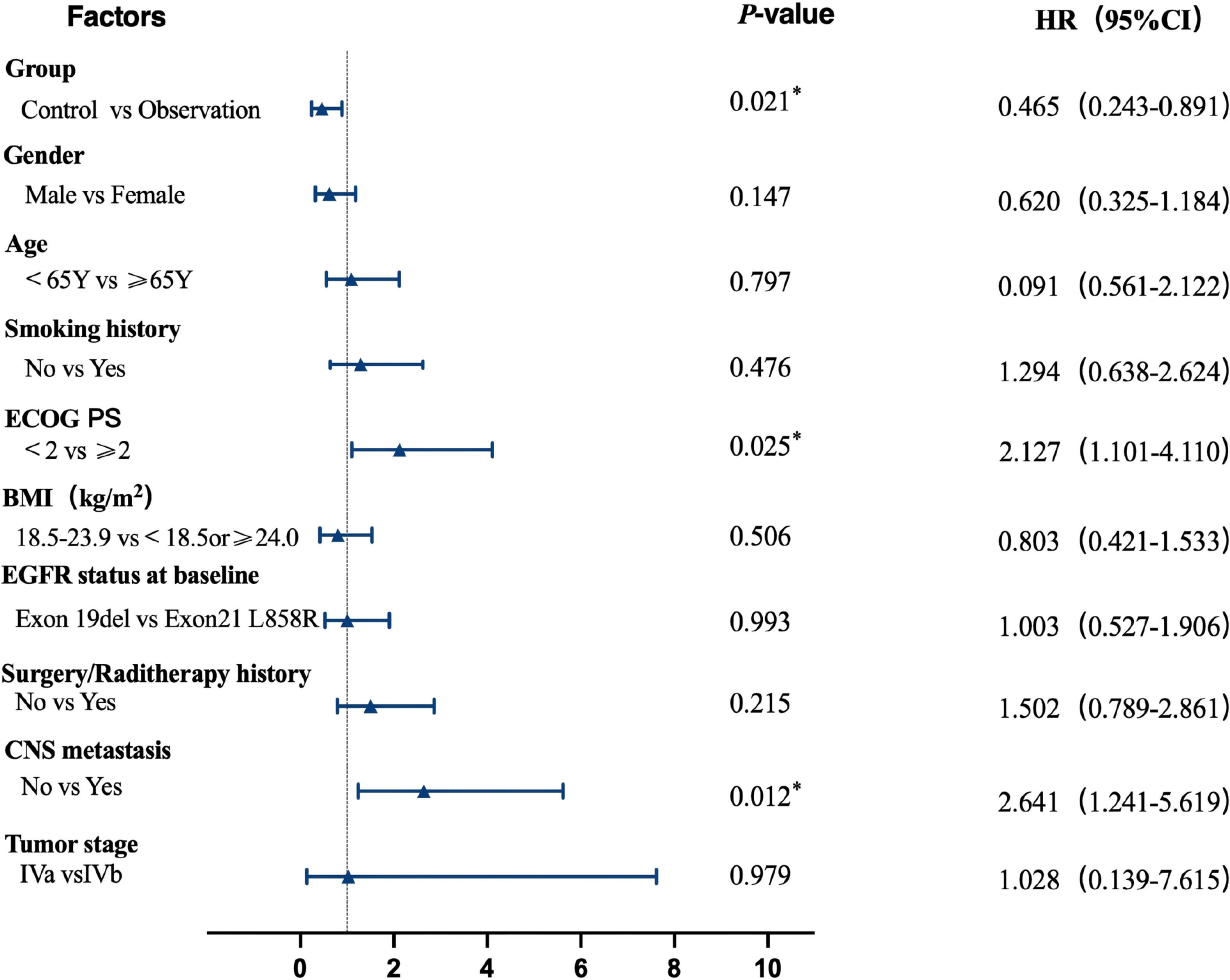
Single-factor analysis. *indicates significant correlation at the 0.05 level (two-tailed).

To explore potential prognostic factors affecting patient outcomes, univariate Cox regression analysis was conducted on relevant clinical characteristics. Results indicated that grouping in this study (*p* = 0.021), CNS metastasis (*p* = 0.012), and ECOG performance status (*p* = 0.025) may be associated with prognosis in patients with advanced NSCLC resistant to third-generation EGFR-TKIs. See **Table 2**, **Figure 3**.

**Table 2:** Analysis of factors affecting PFS.

Factors	Analysis of single factors		Multiplicity	
	*p*-Value	HR (95% CI)	*p*-Value	HR (95% CI)
Group	0.021^*^	0.465 (0.243–0.891)	0.005*	0.383 (0.196–0.750)
Control group vs observation group				
Gender	0.147	0.620 (0.325–1.184)		
Male vs female				
Age	0.797	1.091 (0.561–2.122)		
<65 vs ≥65 years				
Smoking history	0.476	1.294 (0.638–2.624)		
No vs yes				
ECOG PS	0.025^*^	2.127 (1.101–4.110)	0.038*	2.016 (1.040–3.908)
<2 vs ≥2				
BMI (kg/m^2^)	0.506	0.803 (0.421–1.533)		
18.5–23.9 vs <18.5 or ≥24.0				
EGFR status at baseline	0.993	1.003 (0.527–1.906)		
Exon 19del vs Exon21 L858R				
Surgery/radiotherapy history	0.215	1.502 (0.789–2.861)		
No vs yes				
CNS metastasis	0.012^*^	2.641 (1.241–5.619)	0.011*	2.688 (1.253–5.769)
No vs yes				
Tumor stage	0.979	1.028 (0.139–7.615)		
IVa vs IVb				

PFS, progression-free survival; ECOG PS, Eastern Cooperative Oncology Group performance status; BMI, body mass index; EGFR, epidermal growth factor receptor; CNS, central nervous system.

* indicates significant correlation at the 0.05 level (two-tailed).

**Figure 4:**
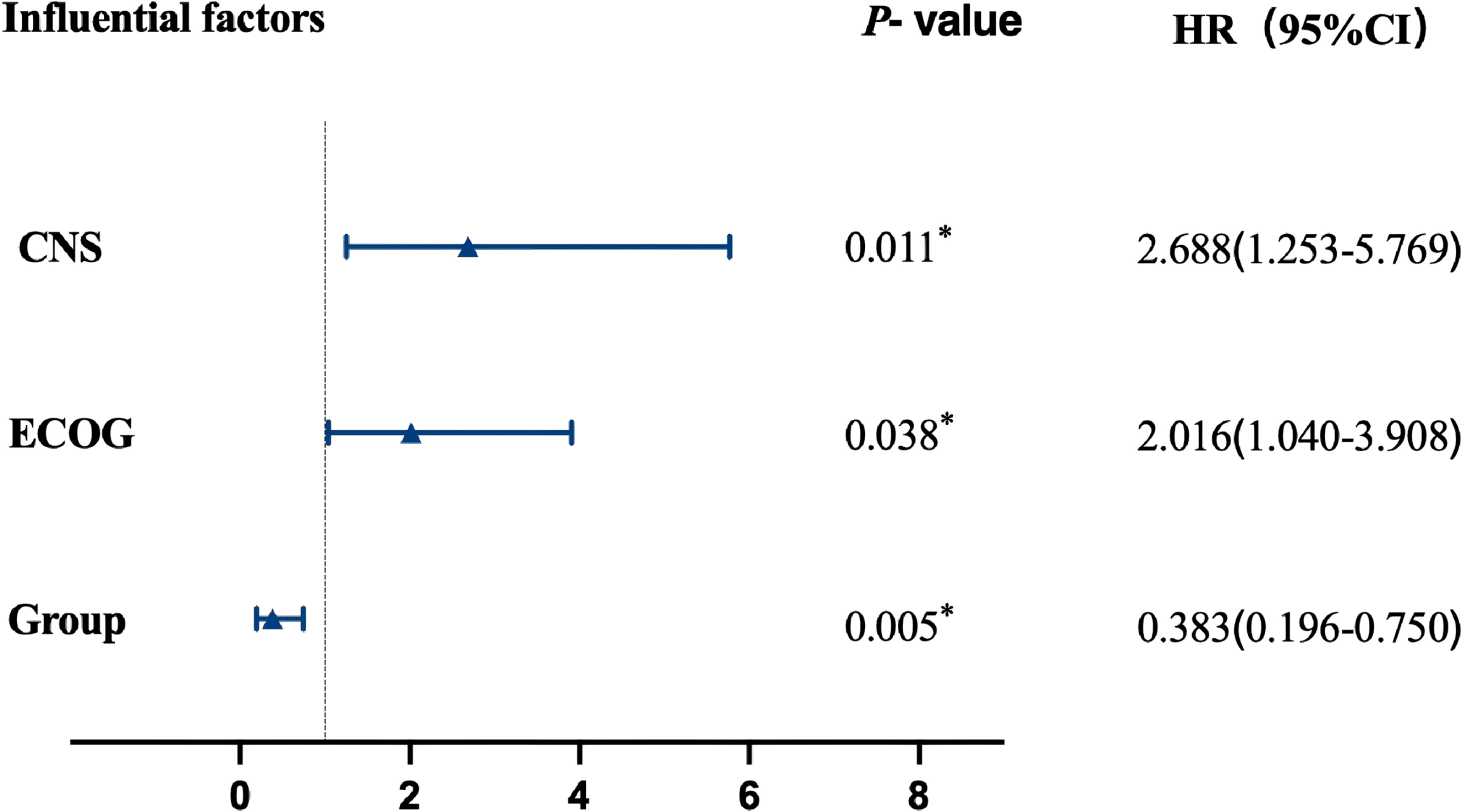
Multifactor analysis. *indicates significant correlation at the 0.05 level (two-tailed).

Based on the results of the univariate analysis, statistically significant variables were incorporated into a multivariate Cox proportional hazards model for further analysis. The results demonstrated that ECOG performance status ≥2 (HR = 2.016, 95% CI: 1.040–3.908, *p* = 0.038) and presence of CNS metastases (HR = 2.688, 95% CI: 1.253–5.769, *p* = 0.011) were independent risk factors affecting patient PFS. Receiving double-dose furmonertinib was an independent protective factor for PFS (HR = 0.383, 95% CI: 0.196–0.750, *p* = 0.005). See **Figure 4**, **Table 2**.

### 3.4 Survival analysis of the CNS metastasis subgroup

**Figure 5:**
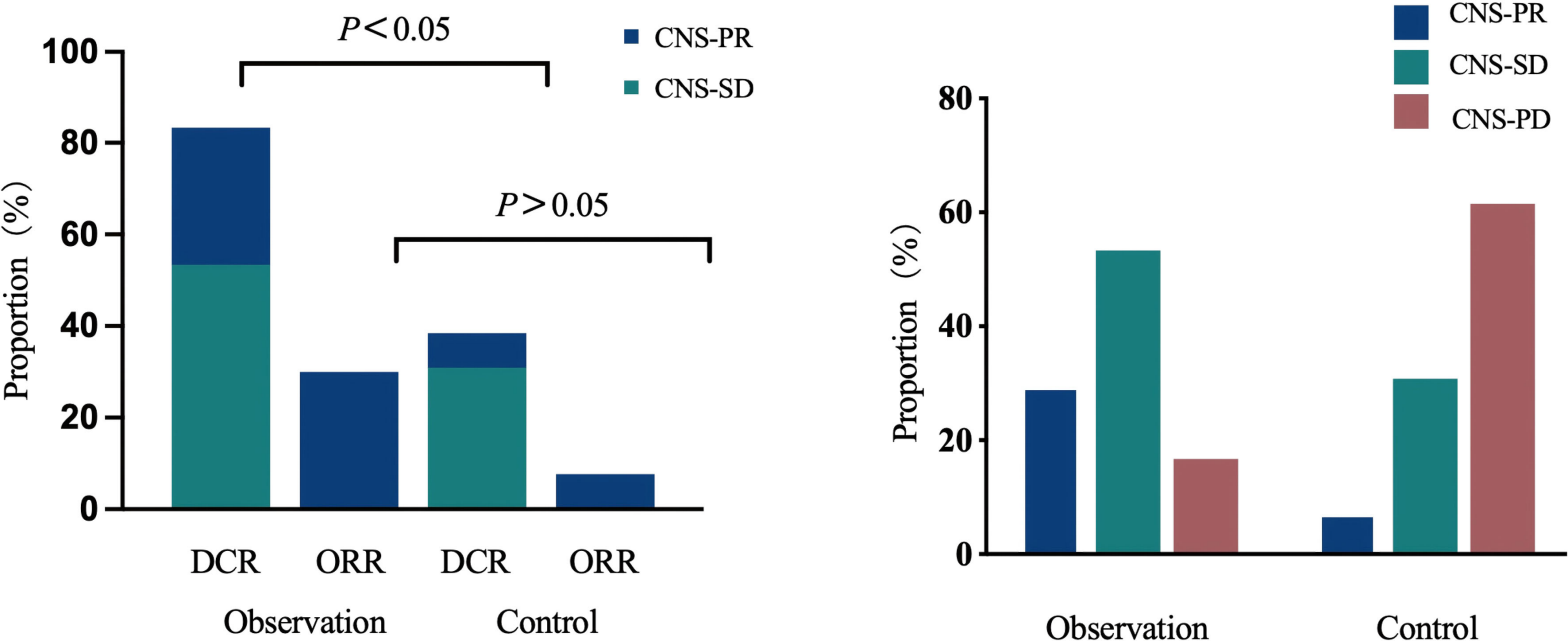
Comparison of optimal treatment outcomes for CNS metastases. CNS, central nervous system.

Local efficacy assessment of CNS lesions in patients with baseline CNS metastases was conducted in both groups, with no cases achieving CNS-CR in either group. Efficacy was assessed in both groups according to RECIST 1.1 criteria. Among the 30 patients with CNS metastases in the observation group, nine achieved CNS-PR, 16 achieved CNS-SD, and five had CNS-PD, yielding a CNS-ORR of 30.0% and a CNS-DCR of 83.3%. Among the 13 patients with CNS metastases in the control group, one achieved CNS-PR, three achieved CNS-SD, and eight had CNS-PD. The CNS-ORR was 7.7%, and the CNS-DCR was 38.5%. See **Table 3**. The CNS-ORR and CNS-DCR rates were higher in the observation group than in the control group. A statistically significant difference was observed between CNS-DCR rates (83.3% vs 38.5%, *p* = 0.003), while no significant difference was found for CNS-ORR (30.0% vs 7.7%, *p* = 0.112). See **Figure 5**.

**Table 3:** Comparison of optimal treatment outcomes for CNS metastases.

	Observation group (n = 30)	Control group (n = 13)	*χ* ^2^	*p*-Value
PR (n)	9	1		
SD (n)	16	4		
PD (n)	5	8		
ORR	30.0%	7.7%	2.529	0.112
DCR	83.3%	38.5%	8.658	0.003^*^

CNS, central nervous system; PR, partial response; ORR, objective response rate; DCR, disease control rate.

*indicates significant correlation at the 0.05 level (two-tailed).

**Figure 6:**
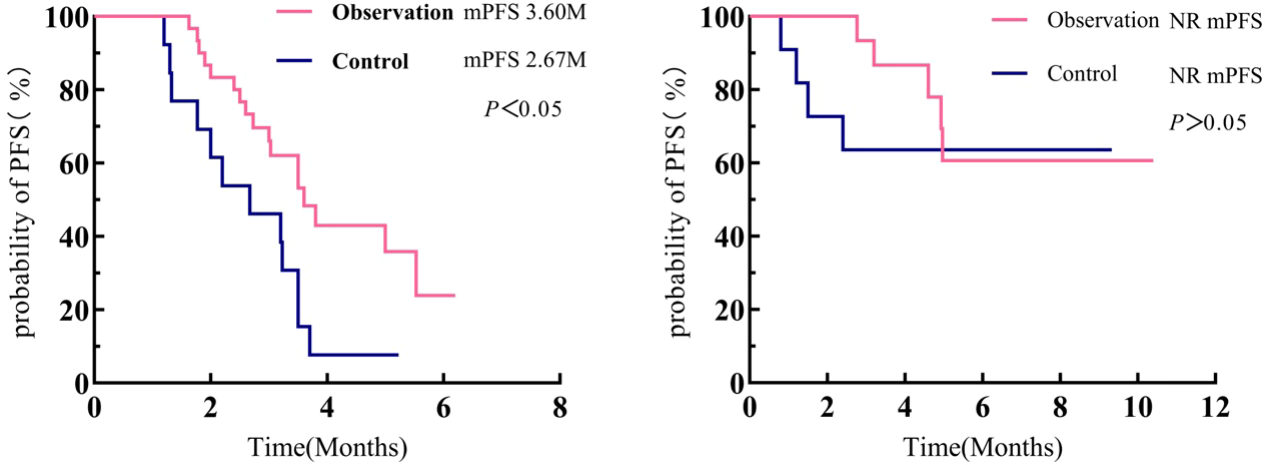
Comparison of survival curves for patients with CNS metastasis versus those without CNS metastasis. CNS, central nervous system.

Patients in the observation and control groups were stratified into CNS metastasis-positive and CNS metastasis-negative subgroups based on the presence or absence of CNS metastasis at baseline. Results showed that among patients with CNS metastases, the observation group had an mPFS of 3.60 months (95% CI: 2.813–4.387), while the control group had an mPFS of 2.67 months (95% CI: 1.261–4.079). A statistically significant difference existed between the two groups (*χ*^2^ = 6.332, *p* = 0.012). Among patients without CNS metastasis, mPFS was not reached in either the observation group or the control group (*χ*^2^ = 0.973, *p* = 0.324). See **Figure 6**.

### 3.5 Safety analysis

**Figure 7:**
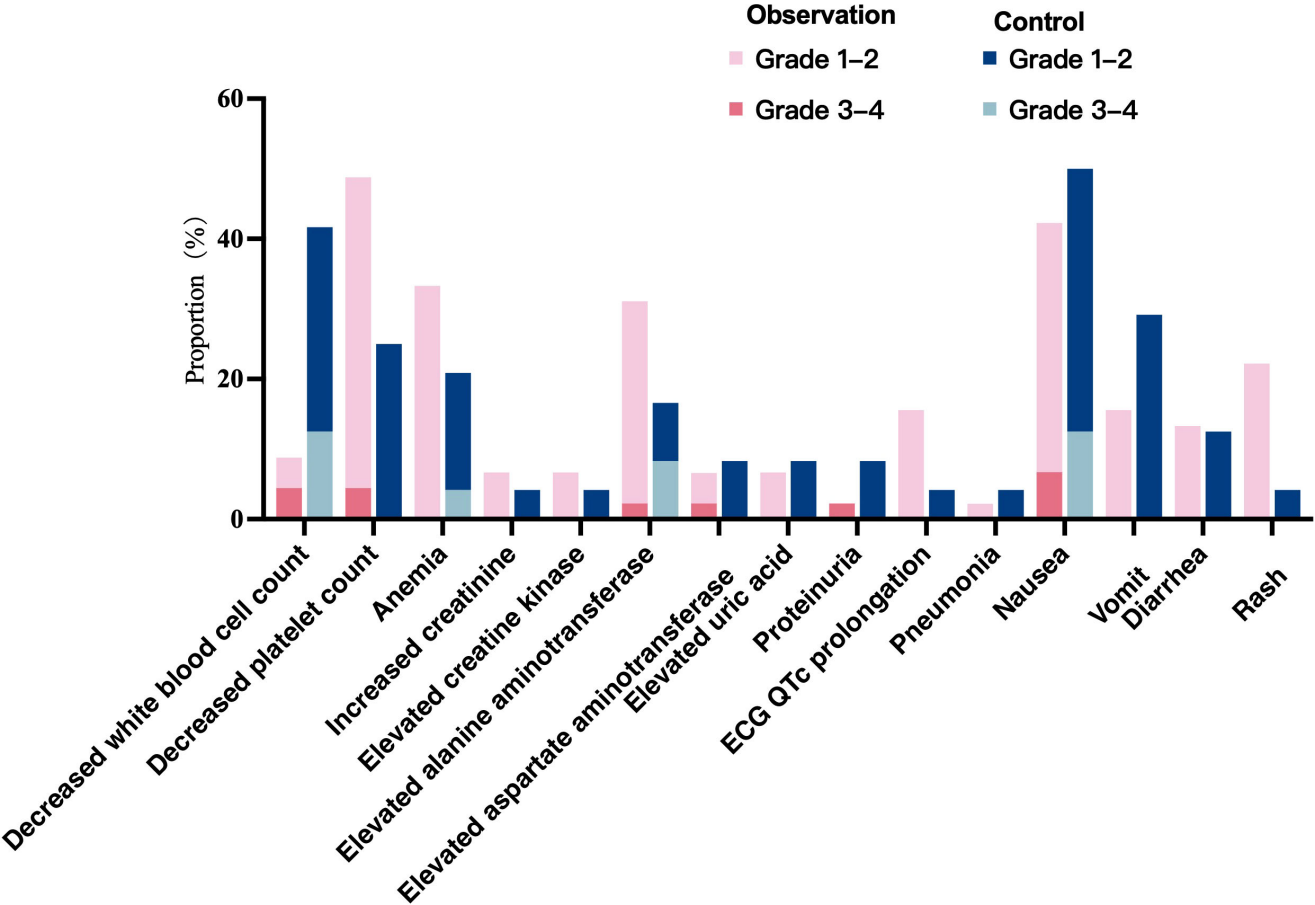
Treatment-related adverse events.

No Grade 5 treatment-related AEs were observed in either group during the study, and no serious fatal or new-onset adverse reactions occurred. In the observation group, the overall incidence of treatment-related AEs was 66.7%. The most common adverse events were thrombocytopenia, nausea, anemia, and elevated transaminases, with most being Grade 1–2 and Grade 3–4 being rare, occurring at a rate of 15.6%. The overall treatment-related AE incidence in the control group was 70.8%, with the most common being nausea, leukopenia, vomiting, and thrombocytopenia—all typical adverse reactions to chemotherapy drugs. Grade 3–4 AEs occurred in 16.7% of cases. There was no statistically significant difference in the overall incidence of treatment-related AEs between the two groups (*p* > 0.05). See **Table 4**, **Figure 7**.

**Table 4:** Treatment-related adverse events.

Treatment-related AEs	Observation group (n = 45)		Control group (n = 24)	
	n (%)	Grade 3–4 n (%)	n (%)	Grade 3–4 n (%)
Total	30 (66.7)	7 (15.6)	17 (70.8)	4 (16.7)
Decreased white blood cell count	4 (8.9)	2 (4.4)	10 (41.7)	3 (12.5)
Decreased platelet count	21 (46.7)	1 (2.2)	6 (25.0)	0 (0)
Anemia	15 (33.3)	0 (0)	5 (20.8)	1 (4.2)
Increased creatinine	3 (6.7)	0 (0)	1 (4.2)	0 (0)
Elevated creatine kinase	3 (6.7)	0 (0)	1 (4.2)	0 (0)
Elevated alanine aminotransferase	14 (31.1)	1 (2.2)	4 (16.7)	2 (8.3)
Elevated aspartate aminotransferase	3 (6.7)	1 (2.2)	2 (8.3)	0 (0)
Elevated uric acid	3 (6.7)	0 (0)	2 (8.3)	0 (0)
Proteinuria	1 (2.2)	1 (2.2)	2 (8.3)	0 (0)
ECG QTc prolongation	7 (15.6)	0 (0)	1 (4.2)	0 (0)
Pneumonia	1 (2.2)	0 (0)	1 (4.2)	0 (0)
Nausea	19 (42.2)	3 (6.7)	12 (50.0)	3 (12.5)
Vomit	7 (15.6)	0 (0)	7 (29.2)	0 (0)
Diarrhea	6 (13.3)	0 (0)	3 (12.5)	0 (0)
Rash	10 (22.2)	0 (0)	1 (4.2)	0 (0)

AEs, adverse events.

## 4 Discussion

Activating mutations in the EGFR tyrosine kinase domain represent one of the most common types of gene mutations in NSCLC that serve as therapeutic targets. Research on targeted drugs for EGFR gene mutations has advanced rapidly, with drugs targeting this mutation site now progressing to the third generation. This has benefited countless patients harboring EGFR gene mutations, and fourth-generation EGFR-TKIs are currently undergoing clinical trials (3). Third-generation EGFR-TKIs have achieved breakthrough progress in treating patients with CNS metastases, thereby gaining widespread clinical recognition. Furmonertinib, as China’s independently developed third-generation EGFR-TKI, retains the unsaturated acrylamide bond and pyrimidine ring while incorporating a unique trifluoroethoxy pyridine structure, thereby exhibiting enhanced selectivity (6, 15). Results from the FURLONG study indicate that the mPFS for first-line furmonertinib treatment in patients with locally advanced or metastatic NSCLC harboring classic EGFR mutations was 20.8 months, marking the first EGFR-TKI monotherapy to achieve a median progression-free survival exceeding 20 months in a frontline setting. Additionally, the study demonstrated reduced disease progression rates and mortality risks among patients with central nervous system metastases (13). Moreover, in dose-escalation trials, furmonertinib demonstrated a superior therapeutic window, exhibiting tolerable safety profiles across doses ranging from 40 to 240 mg (5, 16). This also establishes the foundation for the feasibility and safety of using 160 mg furmonertinib as the therapeutic dose in the observation group of this study. The results of the FIRM prospective clinical trial indicate that the ORR for patients receiving double-dose furmonertinib treatment was 75.8%, with a DCR of 87.9% and an mPFS of 20.2 months. Among patients with baseline brain metastases, ORR reached 80.0%, DCR reached 100.0%, mPFS was not reached, and the incidence of ≥Grade 3 adverse events was only 6.1% (17). Existing studies on the clinical efficacy of double-dose furmonertinib in treating osimertinib-resistant advanced NSCLC patients still lack substantial real-world data support. Furthermore, real-world research findings regarding brain metastases remain insufficient. Therefore, this study aims to investigate the clinical efficacy and safety of double-dose furmonertinib in treating osimertinib-resistant advanced NSCLC patients. All enrolled patients had prior osimertinib treatment history. Through retrospective analysis, we evaluated the clinical efficacy and safety of furmonertinib in drug-resistant advanced NSCLC patients, and the efficacy difference between the double-dose furmonertinib group and the chemotherapy group. Results indicated that the double-dose furmonertinib group significantly improved DCR and prolonged mPFS. Double-dose furmonertinib treatment emerged as an independent protective factor influencing prognosis in this patient population. This further validates the efficacy of double-dose furmonertinib for treating osimertinib-resistant advanced NSCLC patients, offering an effective therapeutic option for this group.

In recent years, high-dose EGFR-TKI treatment strategies have demonstrated potential in overcoming drug resistance. Furmonertinib, a highly selective and irreversible third-generation EGFR-TKI, has demonstrated a favorable safety profile and antitumor activity in preclinical studies and early-phase clinical trials when administered as a double-dose or higher-dose regimen (18). In this study, local efficacy was assessed for CNS metastases in patients with CNS metastases. Results indicated a CNS-ORR of 30.0% in the double-dose furmonertinib group, and CNS-DCR reached 83.3%, demonstrating the superior control efficacy of double-dose furmonertinib against CNS metastases. Furthermore, survival analysis revealed significantly prolonged mPFS in the double-dose furmonertinib group among patients with CNS metastases (3.60 vs 2.67 months, *p* < 0.05), although the CNS response rate was lower than in the aforementioned study. This discrepancy may be related to the administered dose. Future studies may explore higher doses of furmonertinib for efficacy and CNS metastasis control in patients with third-generation EGFR-TKI-resistant advanced NSCLC, while monitoring treatment-related AE incidence. Although no statistically significant difference in survival analysis was observed between the two groups in patients without baseline CNS metastases, neither group achieved mPFS. This may be attributed to the short follow-up duration and limited sample size. Future large-scale prospective studies are needed to further investigate this finding. It is believed that with ongoing research and continuous innovation in detection technologies, more patients will benefit in the future.

Regarding safety, in the Phase III FURLONG study, common adverse reactions in the furmonertinib group included elevated transaminases, diarrhea, and decreased white blood cell count. The incidence of treatment-related AEs of Grade 3 or higher was 11% (19). In the FAVOUR study, 240 mg of furmonertinib was administered to treat advanced NSCLC with EGFR ex20ins mutations. The most common adverse reaction observed during monitoring was diarrhea, predominantly mild to moderate in severity, with overall reliable safety (17). In this study, the most common adverse reactions in the double-dose furmonertinib group included thrombocytopenia, nausea, anemia, and elevated transaminases, which may be related to the concomitant use of chemotherapy agents in some patients. The overall adverse reaction incidence rate was 66.7%. No new or fatal adverse reactions were observed. Overall, patients tolerated the treatment well. Grade 3–4 adverse events improved after symptomatic treatment, with no instances of dose reduction or discontinuation.

In summary, this study demonstrates that double-dose furmonertinib treatment offers significant clinical benefits for patients with advanced NSCLC resistant to osimertinib, with manageable treatment-related adverse events. It also exhibits favorable therapeutic effects on local control of CNS metastases and patient survival, providing evidence for treatment options in patients with advanced NSCLC after osimertinib resistance. This study has limitations. First, as a single-center retrospective study, data and selection biases are unavoidable (e.g., all enrolled patients had adenocarcinoma, and a high proportion were stage IVb patients). Second, the small sample size, short follow-up duration, and significant disparity in patient numbers between the observation and control groups may limit the interpretability of results (e.g., mPFS was not reached in patients without CNS metastases in either group). As this was a single-center study, every effort was made to consecutively enroll all eligible patients during the study period to ensure sample representativeness. Third, treatment regimens varied among patients in the observation group, with not all receiving platinum-based dual-agent chemotherapy. This heterogeneity may have influenced outcomes. Fourth, we did not have access to post-progression tumor tissue or plasma samples to assess known resistance alterations. Given that different resistance mechanisms may differentially influence the efficacy of double-dose furmonertinib, the absence of this information limits our ability to interpret the findings in depth. Future validation requires larger-scale data, prospective studies, and extended follow-up periods.

## 5 Conclusion

In patients with osimertinib-resistant advanced NSCLC, the regimen comprising double-dose furmonertinib yielded promising efficacy, accompanied by an acceptable safety profile and overall tolerable treatment-related adverse events. In patients with osimertinib-resistant advanced NSCLC and CNS metastases, double-dose furmonertinib significantly prolongs PFS and demonstrates marked local control of CNS metastases.

## Data Availability

The raw data supporting the conclusions of this article will be made available by the authors, without undue reservation.
